# Effect of Contact Conditions on Frictional Characteristics of Low-Carbon Deep-Drawn Steel Sheets

**DOI:** 10.3390/ma19061199

**Published:** 2026-03-18

**Authors:** Tomasz Trzepieciński, Sherwan Mohammed Najm, Valentin Oleksik, Mihaela Oleksik

**Affiliations:** 1 Faculty of Mechanical Engineering and Aeronautics, Rzeszów University of Technology, al. Powstańców Warszawy 8, 35-029 Rzeszów, Poland; 2 Department of Fuel and Energy Engineering Techniques, College of Oil & Gas Techniques Engineering—Kirkuk, Northern Technical University, Kirkuk 36001, Iraq; sherwan@ntu.edu.iq; 3 Faculty of Engineering, “Lucian Blaga” University of Sibiu, Victoriei Bd. 10, 550024 Sibiu, Romania; valentin.oleksik@ulbsibiu.ro (V.O.); mihaela.oleksik@ulbsibiu.ro (M.O.)

**Keywords:** deep drawing, friction, sheet metal forming, steel sheet

## Abstract

Friction in sheet metal forming processes is a key factor determining the material flow behavior and surface quality of products. Controlling friction conditions is crucial for ensuring the stability of the forming process. This article focuses on evaluating the influence of strip sample orientation, tool surface roughness, and contact forces on the coefficient of friction in the strip drawing test. Low-carbon, deep-drawing-quality steel sheets produced by rolling were used as the test material. Due to the complex influence of numerous parameters on the coefficient of friction, analysis of variance (ANOVA) was employed to evaluate the experimental results. A two-factor interaction model was used to analyze the data from the strip drawing test. An adequate precision of approximately 104.74 and coefficients of determination of R^2^ = 0.9367, an adjusted R^2^ = 0.9350, and a predicted R^2^ = 0.9331 indicated that the regression model was sufficiently fitted to provide reliable predictions. It was found that the influence of sheet orientation on the coefficient of friction, under a varying load force, was minor; the difference in the CoF between the two sample orientations did not exceed about 0.01. On the other hand, among all the parameters of the strip drawing test, the load force was the decisive factor affecting the CoF. A trend was observed in which the coefficient of friction increased with a decreasing average roughness of the countersamples and load force.

## 1. Introduction

Sheet metal forming (SMF) is one of the leading plastic-forming methods. Sheet-metal-forming methods are crucial in the automotive and household appliance industries because they enable economical, rapid, and precise production of thin-walled components with complex shapes [1]. Deep drawing enables the mass production of repeatable parts at relatively low unit costs. As a result of plastic deformation, the workpiece undergoes the work-hardening phenomenon, which increases the stiffness and strength of the material [2]. Low-alloy steel sheet metals intended for plastic forming should demonstrate appropriate formability [3].

The deformation of sheet metal depends on ensuring there are appropriate friction conditions between the surfaces of the workpiece and the stamping tools employed [4]. Resistance to motion between two contacting surfaces is defined by the value of the coefficient of friction (CoF) [5]. The CoF depends on many parameters, including the type of friction pair, lubrication conditions, contact pressure, sliding speed, and the surface roughness of the friction pair surfaces [6]. Friction is the main phenomenon limiting material flow during deformation, intensifying changes in the surface topography of the sheet metal and causing accelerated tool wear [7,8]. For this reason, sheet-forming processes are carried out mainly under lubricated conditions using lubricants adapted to the friction conditions [9,10]. These are usually synthetic lubricants, but research is also being conducted on the use of bio-lubricants of natural origin [11,12]. The optimality of the forming process is dependent on determining the value of the CoF of the sheets intended for processing. There are many methods for determining the CoF in sheet-metal forming, but the most commonly used is the strip drawing test, which involves pulling a sheet strip between two countersamples with flat [13] or cylindrical [14] working surfaces.

Understanding friction under specific forming conditions makes it possible to optimize production processes by allowing one to select the appropriate lubricant, prepare tool surfaces, define forming conditions, reduce tool wear, and improve the surface quality of components [15,16]. Experimental studies are essential for understanding the phenomenon of friction and allow the influence of various factors to be evaluated. Tagije et al. [17] found that during strip drawing testing of a DC04 steel sheet, under both dry and lubricated friction conditions, the CoF decreases as the nominal contact pressure increases. The effectiveness of lubrication strongly depends on oil viscosity and contact pressure—at higher pressure, the lubricant film is less effective, leading to a smaller reduction in CoF [13]. Changes in sheet surface topography are directly related to friction mechanisms such as the flattening of asperities and surface roughening [18]. Kohutiar et al. [19] observed the formation of oxidation layers during dry friction and indicated differences in the CoF depending on the geometry of the movement in the ball-on-disc method. These authors also reported combined wear mechanisms (adhesive and abrasive) in 30CrNiMo8/G40 steel contacts and analyzed the influence of oxygen and carbon distribution on the reduction in the CoF, which was explained by the effect of so-called “permanent micro-lubrication”. Vollertsen and Hu [20] found that the CoF decreases with an increase in punch diameter in the sheet metal forming of axisymmetric components as a result of the tribological size effect. Jewvattanarak et al. [21] used the strip drawing test to evaluate the lubrication quality of different lubricants. These authors reported that the CoF decreases with an increasing Simmerfield number, which is a crucial dimensionless quantity in the analysis of lubrication. Shrivastava and Digavalli [22] investigated the effect of important process variables, namely, drawing speed and contact pressure, on the CoF at elevated forming temperatures. The results obtained from the strip drawing tests indicated that the CoF increased with a rising temperature and a decreasing drawing speed. Makhkamov [23] found that lubrication helps minimize the effect of the directionality of tool surface topography on the CoF. De Oliveira Lopes et al. [24] investigated how contact pressure and sliding speed influence the tribological performance of cold-work tool steels in the strip drawing test. Contact pressure had a more pronounced effect on the wear and tribological behavior of cold-work tool steels than sliding speed.

Low-carbon steel sheets are produced through a rolling process, which introduces a directional orientation of the grains in the material. The resulting anisotropy of mechanical properties and specific topography change the conditions of the deformation of surface asperities during friction. Among the many parameters influencing the CoF in sheet metal forming, sheet orientation remains insufficiently addressed. Therefore, further in-depth studies are necessary to clarify the effect of this parameter on friction conditions. For this reason, the aim of the experimental research presented in this work was to examine the influence of strip sample orientation on the CoF when drawing-quality steel sheets come into contact with 145Cr6 cold-work tool steel. This influence was investigated under varying contact forces and degrees of tool surface roughness. Due to the complex impact of numerous parameters on the CoF, analysis of variance (ANOVA) was used to evaluate the experimental results. The novelty of this study lies in its systematic experimental and analytical assessment of the influence of strip orientation on frictional behavior in a material pair commonly used in industrial sheet metal forming, providing insights directly applicable to sheet-metal-forming process design.

## 2. Materials and Methods

### 2.1. Test Material

Friction tests were performed on low-carbon steel sheets with varying degrees of formability: 1 mm thick drawing quality (DQ) steel sheet, 0.8 mm thick deep drawing quality (DDQ) steel sheet, and 1 mm thick extra-deep drawing quality (EDDQ) steel sheet. The tested sheets were manufactured in accordance with the Polish standard PN-87/H-92143 [25]. The grades DQ, DDQ, and EDDQ correspond to DC01, DC03, and DC04 steel sheets, respectively, in accordance with the standard EN 10130:2009 [26]. The mechanical properties of the tested sheets were determined in a uniaxial tensile test in accordance with standard ISO 6892-1 [27]. The tests were performed on samples cut longitudinally (orientation 0) and transversely (orientation 90) to the sheet-rolling direction. Based on three repetitions, average values of the following parameters were determined: elongation (A), yield strength (YS), ultimate tensile strength (UTS), work-hardening coefficient (K), and work-hardening exponent (n) (Table 1). The values of the basic spatial parameters of the tested sheets, determined using the Alicona InfiniteFocus instrument, are presented in Table 2. Average roughness Sa and root-mean-square roughness Sq are the basic parameters used to characterize the roughness of the sheets. Kurtosis Sku and skewness Ssk are statistical measures used to characterize the shape of the elevation distribution of surfaces subjected to friction.

### 2.2. Strip Drawing Test

**Figure 1:**
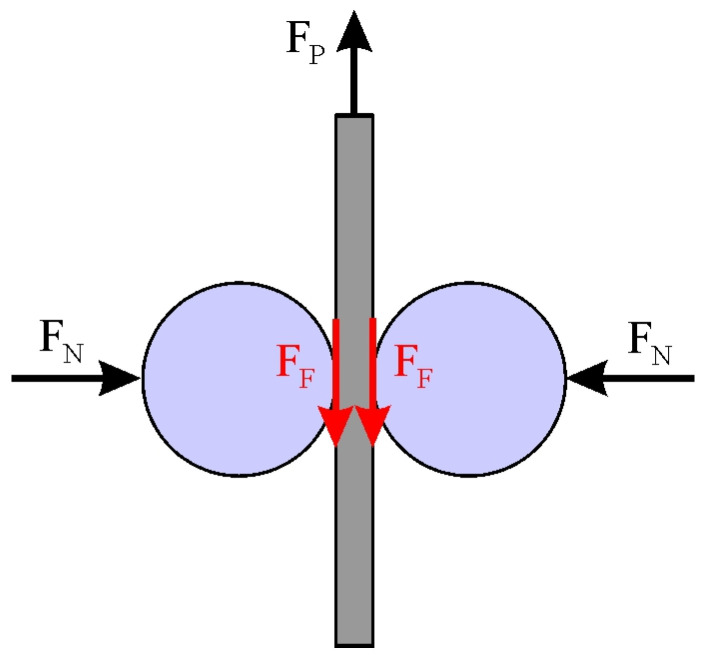
Scheme of strip drawing test.

The strip drawing test (Figure 1) is used to simulate friction conditions in the blankholder zone in SMF operations. In this zone, under the influence of the hold-down force F_B_, the frictional force F_F_ takes effect. The proportionality factor between these forces is the CoF. According to Amontons’ law of friction, the coefficient of friction is [28]

(1) $$CoF=\frac{F_{F}}{F_{B}}$$

The pulling force F_P_ balances the frictional resistance on the contact surfaces between the sheet strip and the countersamples (Figure 1); therefore,

(2) $$F_{P}=2F_{F}$$

After being rearranged, Equation (2) can be written as

(3) $$F_{F}=\frac{F_{P}}{2}$$

By substituting Equation (3) into Equation (1) and considering that the normal force F_N_ in the strip drawing test directly corresponds to the force F_B_ [28], we obtain the following:

(4) $$CoF=\frac{F_{P}}{{2F}_{N}}$$

Over the last few decades, many authors have used Equation (4) to determine the CoF in the strip drawing test, including Adamus et al. [9], Guo et al. [13], Lee et al. [29], Roizard et al. [30,31], and Więckowski et al. [28], among others.

### 2.3. Friction Testing

**Figure 2:**
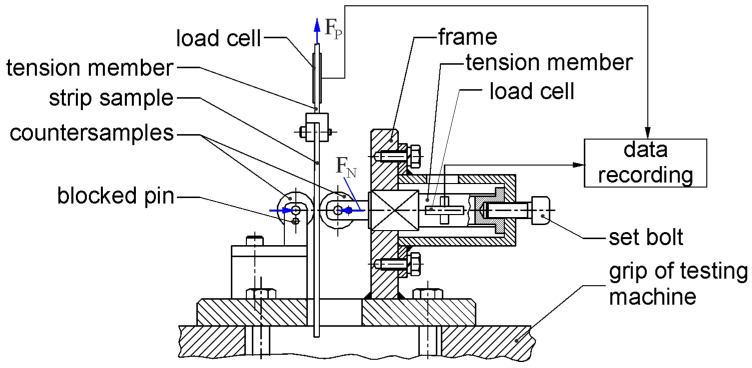
Schematic diagram of the friction tester.

Experimental tests intended to determine the coefficient of friction were conducted using the strip drawing test, which is commonly used for tribological characterization of sheets used in sheet metal forming. The self-made laboratory tester employed (Figure 2) consists of a body to which two cylindrical countersamples with a diameter of 20 mm are attached. The countersamples were made of cold-worked 145Cr6 tool steel.

The tester body was mounted in the lower grip of a universal testing machine Zwick/Roell Z100 (ZwickRoell GmbH & Co. KG, Ulm, Germany). In the upper grip of the testing machine, strip samples with a width of 20 mm and a length of 200 mm were clamped. The strip samples were cut, similarly to the specimens for the uniaxial tensile test, longitudinally (orientation 0°) and transversely (orientation 90°) to the sheet-rolling direction. The cylindrical countersamples were mounted in a non-rotating configuration. Tests were conducted with a sliding speed of 5 mm/s controlled by the Zwick/Roell machine drive system. The actual drive speed (sliding speed) was controlled and maintained within a maximum deviation of ±0.05% in relation to the speed set in the machine control system [32].

**Figure 3:**
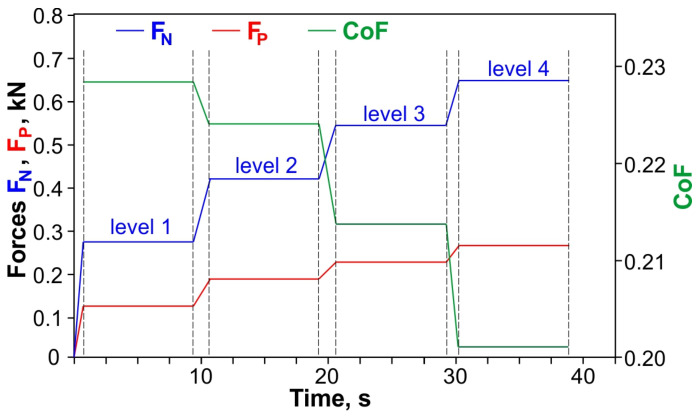
Schematic diagram of changes in values of forces and the coefficient of friction during the friction test for a clamping force variation range of 0–0.7 kN.

The values of both the clamping force F_N_ and the pulling force F_P_ were continuously recorded using an electric resistance strain gauge technique, an 8-channel universal amplifier of HBM’s QuantumX (Hottinger Baldwin Messtechnik GmbH, Darmstadt, Germany) data acquisition system, and a personal computer. The tests were carried out with three repetitions. During each test, the clamping force was gradually increased up to about 2 kN. Figure 3 shows a schematic diagram of the changes in the values of forces and coefficient of friction during the friction test for a clamping force variation range of 0–0.7 kN. Due to the limited length of the strip samples (200 mm), four levels of change (four average CoF values) were obtained on one strip sample (Figure 3). During the tests, different clamping forces were applied for specific sheet grades, sample orientations, and degrees of countersample roughness. The clamping force levels were not identical for all tested sheets. For each friction condition, tests were conducted at 14–16 clamping force levels with a variation range of 0—approximately 2 kN. The different values of the clamping force levels (14–16) result from the desire to provide unbalanced data. Consequently, the dataset has a non-uniform distribution of clamping force. This does not affect the validity of the statistical evaluation because ANOVA can be applied to unbalanced experimental designs, provided that the model assumptions are satisfied. For each F_N_ force level, the average CoF was determined. Based on these average results for the three experiments, the global average value of the friction coefficient was determined according to the following relationship:

(5) $$CoF(average)=\frac{\sum_{n=1}^{k}\left(\frac{F_{P}}{{2F}_{N}}\right)}{k}$$

where F_P_ is pulling force, F_N_ is clamping force, and k = 3 is the number of measured pulling and normal forces for specific level of CoF (Figure 3).

The total number of observations (264) results from the combination of three steel grades, two specimen orientations, three countersample conditions, and 14–16 levels of clamping forces.

The tests were conducted under lubrication conditions using S100 Plus oil (Naftochem^®^ sp. z o.o., Kraków, Poland) with a kinematic viscosity of 360 mm^2^/s. This oil is intended for industrial deep drawing of parts with complex geometries. Before the test, the strip samples and countersamples were cleaned and degreased with acetone. The lubrication conditions were adapted from the study conducted by Payen et al. [33], who investigated friction affecting steel sheets in a strip drawing test. The oil was first applied using a soft brush. Subsequently, the oil layer was regularized with the brush after the excess lubricant was removed. These conditions correspond to the absence of droplet formation on the downward-oriented brush bristles. In the final stage, the specimen was left for 10 min to allow excess lubricant to drain under the effect of gravity. The average quantity of lubricant applied was approximately 7 g/m^2^.

**Figure 4:**
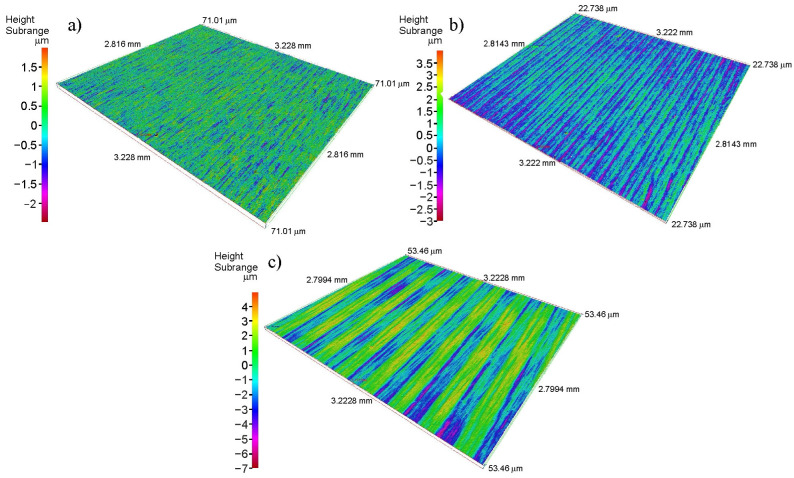
Surface topographies of countersamples characterized by the following average roughness values: (**a**) Ra = 0.32 μm, (**b**) Ra = 0.63 μm and (**c**) Ra = 1.25 μm.

Three sets of countersamples with different degrees of surface roughness were used in the tests. Standard surface finish grades (such as the ISO N grades) were used to characterize the countersamples’ surface roughness: Ra = 0.32 μm, Ra = 0.63 μm, and Ra = 1.25 μm. These parameters, measured along the countersample generatrix, were used as qualitative indicators of surface quality at the countersamples’ production stage. Diagrams of the countersamples’ surfaces and topographies are shown in Figure 4.

Figure 4 shows the surfaces of the countersamples that were produced by machining. The marks visible in the figure originate from the machining of the cylindrical countersamples. According to the order specifications, the countersamples were required to have average surface roughness values of Ra = 0.32 µm, Ra = 0.63 µm, and Ra = 1.25 µm, measured along the generatrix of the cylindrical countersamples (Figure 1), i.e., perpendicular to the direction of the machining marks. This approach facilitated control of the machining parameters, allowing us to obtain the required surface roughness. Areal parameters of the countersamples’ surfaces (Table 3) were also determined using the Alicona InfiniteFocus instrument.

### 2.4. ANOVA

Analysis of variance is used to evaluate the statistical significance of the influence of individual parameters (inputs) and their interactions on the dependent variable measured (response). ANOVA divides the total variability of the data into variability between groups and variability within groups. If the variability between groups is significantly greater than the variability within groups, this indicates that the differences between group means are not random [34]. Variance is a measure of the deviation of empirical values from the arithmetic mean. Table 4 presents the input variables and their variability ranges. Sample orientation, Ra of countersamples, and load forces were the numerical variables considered. Sample orientation was numerically encoded for regression convenience, whereas the interpretation of the results for this study was limited to the two tested levels, 0° and 90°. Type of steel sheet metal was a categorical variable.

Statistics pertaining to the response variable are presented in Table 5.

Under friction conditions in SMF processes, the Ra parameter (or its areal equivalent, Sa) is justifiably the most appropriate and universal roughness parameter due to its direct relationship with the character of frictional contact [35]. In sheet-forming operations, contact occurs primarily at the asperity peaks, whose mean height amplitude determines the conditions for the formation of lubricant pockets and lubricant retention. The Ra and Sa parameters, defined as the arithmetic means of height deviations, adequately reflect the overall development of surface topography and thus influence the intensity of abrasion, flattening and lubricant retention. Moreover, average roughness is the most commonly used roughness parameter in industry [36], enabling a direct linkage between laboratory research results and technological practice.

The significance of the individual factors, along with their two-way interactions, was examined at a 95% confidence level (*p* < 0.05). Significant model terms were selected using backward elimination regression. Terms with *p*-values > 0.100 were removed.

## 3. Results and Discussion

When performing analysis of variance, it is necessary, from a statistical standpoint, to assess whether there is a strong correlation between the explanatory variables and the dependent variable (response). If there is some degree of internal correlation between the factors, one of them must be removed, as it will ultimately harm the model.

**Figure 5:**
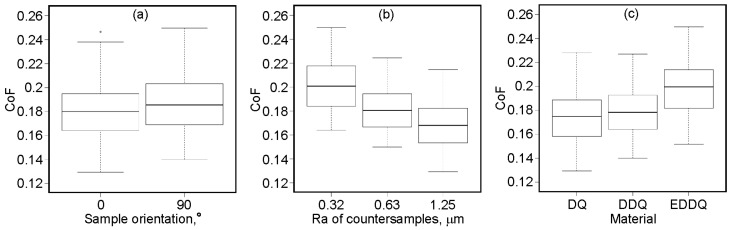
Box plots for (**a**) sample orientation, (**b**) Ra of countersamples, and (**c**) grade of steel sheet metal.

The box plots for all the parameters are approximately the same height (Figure 5). The height of the box corresponds to the interquartile range, which encompasses the middle 50% of observations. Inside each box, there is a horizontal line representing the median value. The median divides the interquartile range into two regions, each containing 25% of the observations. Outliers are represented by points that are more than 1.5 times the interquartile range above the third quartile. The whiskers encompass the 25% of observations with values below the lower quartile and above the upper quartile. In all the cases analyzed, the median divides the boxes into two equal parts; however, analysis of the whisker lengths indicates a slightly right-skewed distribution.

The correlation matrix between input factors and the CoF (Table 6) shows how factors are correlated with each other on a scale from −1 (a negative correlation) to +1 (a positive correlation). There are strong negative correlations between the mean roughness of the countersamples and the CoF (−0.546) and between the normal load and the CoF (−0.655). A negative correlation means that as the value of one variable increases, the value of the other decreases.

The software product Design-Expert (version 12) allows for the automatic selection of an optimal statistical model that explains significant variation without being overly complex or aliased, using criteria such as coefficients of determination, standard deviation, and the predicted residual error sum of squares (PRESS) (Table 7). A preliminary data analysis conducted using Design-Expert suggested that a two-factor interaction (2FI) model would best estimate the effects of the input parameters on the CoF.

The F-value was used to test the significance of adding new model terms to those already included in the model. The model F-value of 541.60 (Table 7) indicates that the mathematical model we developed is significant. The probability that such a large F-value is due to noise is only 0.01%. Model parameters are not significant if the *p*-value is greater than 0.05. In the established ANOVA model, the significant parameters are sample orientation, surface roughness of the countersamples, normal load, and type of sheet metal. The difference between the adjusted and predicted coefficients of determination is smaller than 0.2, indicating that the mathematical model predicts new data with sufficient accuracy. In the model we developed, this criterion was met: the adjusted R^2^ is 0.9350, while the predicted R^2^ is 0.9331 (Table 8). A signal-to-noise ratio (adequacy precision) greater than 4 is desirable. The adequate precision of 104.7431 indicates that the model is sufficiently fitted to provide reliable predictions. Due to its statistical significance and high coefficient of determination (R^2^ = 0.9379), the developed mathematical model can be used to predict the coefficient of friction within the range of the explanatory variables.

**Figure 6:**
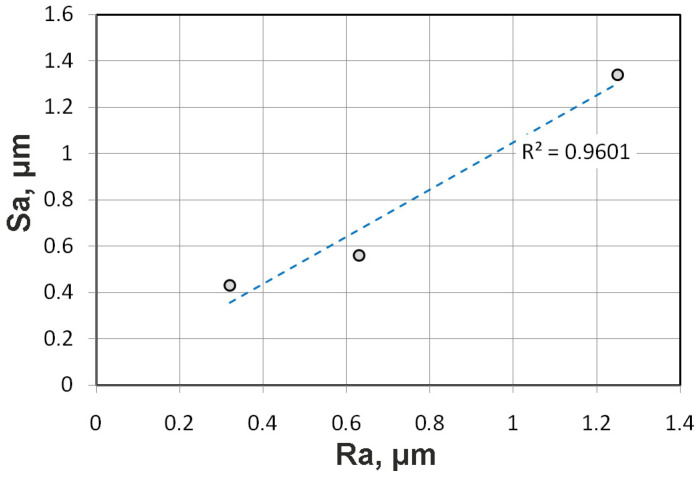
Relationship between the roughness parameters Ra and Sa of the countersamples.

The Ra of the countersamples is a qualitative indicator from the production stage, and Ra is simultaneously used as the principal roughness factor in the regression model. From an industrial point of view, the average roughness Sa may provide a better representation of the contact zone, since contact occurs over the surface of contact. Analyses of variance were also performed for the average roughness parameter, Sa, of the countersamples (Table 3); it is included as parameter B in Table 4. The obtained coefficients of determination R^2^, adjusted R^2^, and predicted R^2^ were 0.8853, 0.8821, and 0.8790, respectively. The coefficients of determination are the primary indicators of the predictive quality of the regression model. Thus, the coefficients of determination for the model including the Ra of the countersamples (Table 4) were more than 5% higher than those for the model including the parameter Sa. Although a strong correlation exists between the Ra and Sa parameters of the countersample surfaces (Figure 6), a comparison of the regression models indicates that the Ra parameter of the countersample surface is more correlated with the CoF than the Sa parameter. Therefore, further analyses were conducted for the model containing the input parameters listed in Table 4.

**Figure 7:**
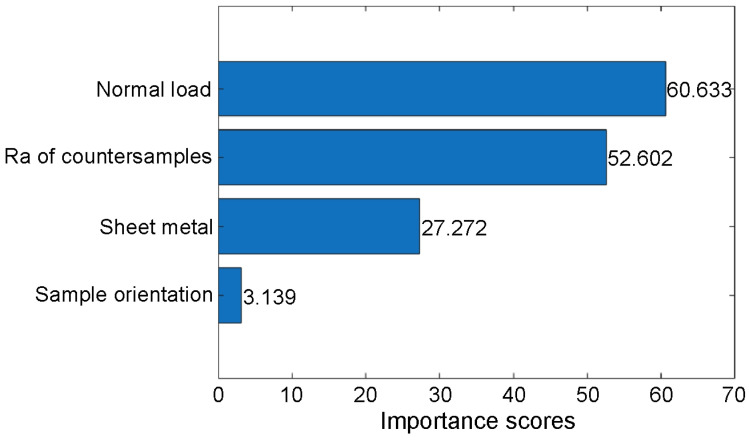
Feature importance scores sorted using F test algorithm.

The importance of each predictor was examined individually using the F test algorithm (fsrftest) built in Matlab R2025a. The fsrftest algorithm examines the significance of each predictor based on the *p*-value of the F-test statistic. Each F-test tests two hypotheses. The first assumes that the response values come from a population with the same mean. The second, competing hypothesis assumes that the population means are not the same. A low *p*-value for the test statistic indicates that the corresponding predictor is significant. However, the result is presented as −log(p), so a high importance score value indicates that the corresponding predictor is significant. Type of sheet metal was considered a categorical feature. The remaining features were numerical. According to the results, the normal load is the most important feature (Figure 7). In order of decreasing importance score, the Ra of countersamples, type of sheet metal, and sample orientation were classified.

Equations expressed in terms of actual factors based on the 2FI polynomial model for the CoF of the DQ, DDQ, and EDDQ steel sheets are presented in Equation (6), Equation (7) and Equation (8), respectively. The equations, expressed in terms of the actual factors, can be used to predict the coefficient of friction for the levels of variation for each factor corresponding to the experimental tests.

(6) $$CoF\left(DQ\right)=0.229337+0.000067\cdot sample\;oriantation\left({}^{\circ}\right)-0.032346\cdot Ra\;of\;countersamples\left(\mu m\right)-0.029525\cdot normal\;load\;(kN)$$

(7) $$CoF\left(DDQ\right)=0.230386+0.000067\cdot sample\;oriantation\left({}^{\circ}\right)-0.029817\cdot Ra\;of\;countersamples\left(\mu m\right)-0.029525\cdot normal\;load\;(kN)$$

(8) $$CoF\left(EDDQ\right)=0.257636+0.000067\cdot sample\;oriantation\left({}^{\circ}\right)-0.039876\cdot Ra\;of\;countersamples\left(\mu m\right)-0.029525\cdot normal\;load\;(kN)$$

In the ANOVA inputs, sample orientation is treated as a continuous numerical variable ranging from 0° to 90°, whereas the experimental design appears to involve only two levels (0° and 90°). Regression models allow for the determination of CoF values for these two levels of sample orientation.

**Figure 8:**
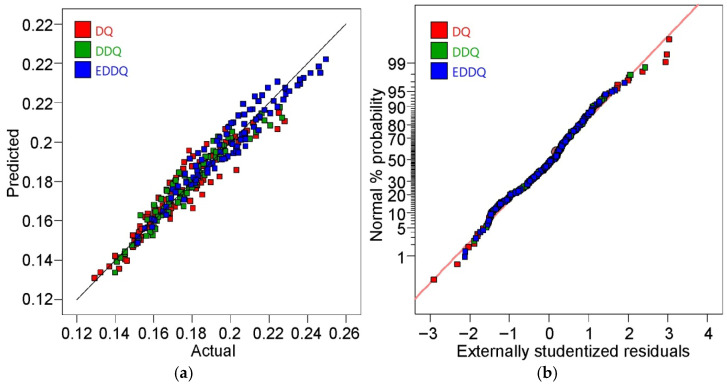
(**a**) Predicted versus actual COF and (**b**) normal probability plot of externally studentized residuals.

**Figure 9:**
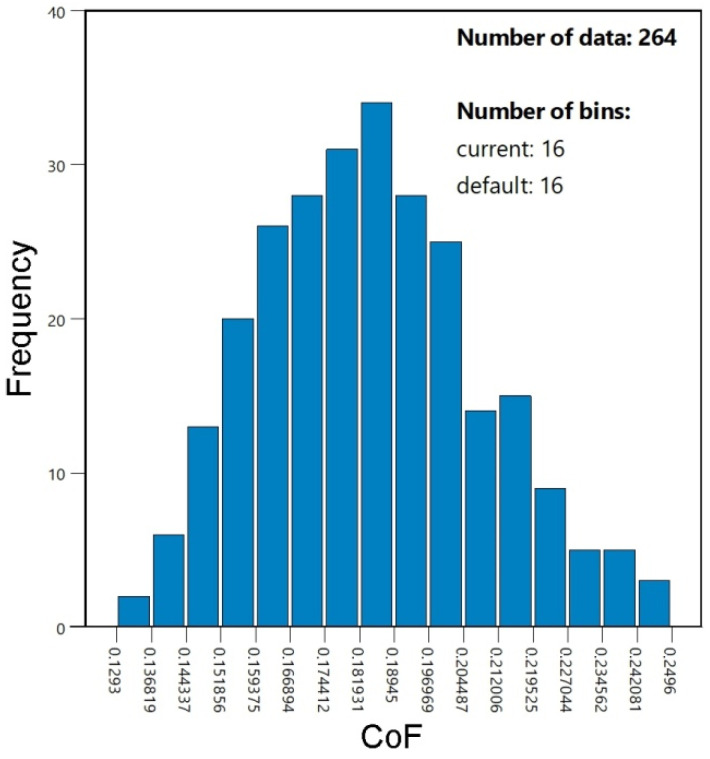
Frequency distribution of CoF differences.

The response values derived from the model, based on the input data introduced into the regression equations, were compared with the experimental values (Figure 8a). The predicted values of the coefficient of friction were distributed proportionally on both sides of the line, representing the ideal prediction; i.e., CoF_predicted_ = CoF_experimental_. A normal probability plot of externally studentized residuals was used to check whether the model errors were normally distributed by plotting the ordered residuals against the expected normal scores (Figure 8b). The shape of the distribution of externally studentized residuals is very close to a straight line, which indicates the normality of their distribution [37]. The normality of the response value distribution is required for performing an analysis of variance on the dependent variable. However, ANOVA tolerates violations of this assumption fairly well. ANOVA can handle data that are not normally distributed (with skewed or kurtotic distributions), with only a minor effect on model errors. As shown in Figure 9, the experimentally determined values of the coefficient of friction exhibit a normal distribution, where the frequency of occurrence decreases with an increase in distance from the mean line.

**Figure 10:**
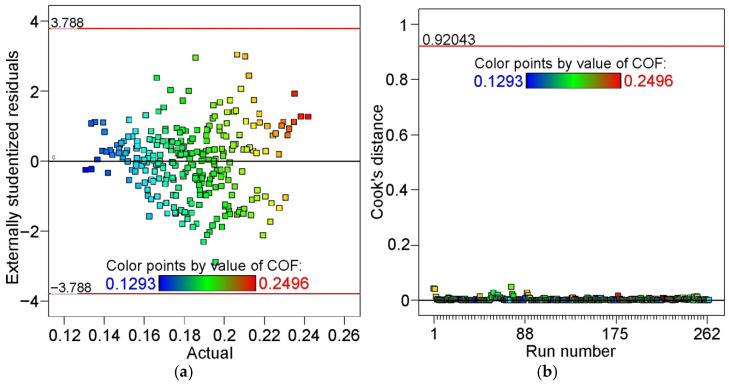
(**a**) ESRs vs. actual CoF and (**b**) Cook’s distance vs. run number.

The randomness of residuals can be assessed by plotting externally studentized residuals (ESRs) against the actual CoF values (Figure 10a). Externally studentized residuals are calculated by removing each point to obtain an independent estimate of error, making extreme values stand out more clearly than raw or internally studentized residuals. The reliability of the model is confirmed by the random scattering of the residuals around the zero line. The lack of systematic distribution confirms the absence of systematic errors or any discernible pattern [38]. All points fall within the limits (±3.788), demonstrating the absence of outliers that could impact the model’s predictions.

Figure 10b shows the relationship between Cook’s distance and CoF. The general equation for Cook’s distance D_i_ for the i-th observation is

(9) $$D_{i}=\frac{{\left(y_{i}-{\hat{y}}_{i}\right)}^{2}}{\mathrm{MSE}\left(1+k\right)}\left[\frac{h_{\mathrm{ii}}}{{\left(1-h_{\mathrm{ii}}\right)}^{2}}\right]$$

where MSE is mean square error, k is the number of terms in the model, y_i_ is is the actual response value for the i-th point, ${\hat{y}}_{i}$ is the predicted response for the i-th point, and h_ii_ is the leverage of the i-th observation.

Cook’s distance evaluates the influence of individual observations on the results yielded by a model, that is, how much the estimated means and model would change if a given observation were removed. In other words, it is essentially the sum of differences in predictions at every point caused by leaving one point out when fitting the model [39]. Figure 10b shows no points that deviate significantly from the rest of the data. The higher the Cook’s distance, the greater the influence of a given observation on the outcome produced by the model. None of the points exceed the threshold value of 0.92043. Therefore, all the points meaningfully affect the model’s response.

The statistical parameters DFFITS (difference of fits) and DFBETAS (standardized difference of the betas) are used to check for outliers in the model. DFFITS (Equation (10)) assesses the change in the estimated value of the dependent variable when a specific observation is removed from the model [40]:

(10) $$\mathrm{DFFITS}\;=\;\frac{{\hat{Y}}_{i}-{\hat{Y}}_{i(i)}}{s_{\left(i\right)}\sqrt{h_{\mathrm{ii}}}}$$

where ${\hat{Y}}_{i}$ is the prediction of the model with all observations accounted for, ${\hat{Y}}_{i(i)}\;$ is a prediction without data point i included in the regression model, s_i_ represents the standard error estimated without the ith point included, and h_ii_ is the leverage value.

In other words, the DFFITS determines how much the fitted values would change if a given observation were removed from the model. The limits for the DFFITS are computed using the following expression:

(11) $$\pm \max\left(1,\;3\sqrt{\frac{p}{n}}\right)$$

where p and n are the number of terms and runs in the model, respectively.

DFBETAS (Equation (11)) estimates the change in the regression coefficient resulting from the removal of a specific variable from the model [40].

(12) $$\mathrm{DFBETAS}=\frac{{\hat{\beta }}_{j}-{\hat{\beta }}_{i,j}}{\sqrt{{\mathrm{MSE}}_{\left(i\right)}\times c_{\mathrm{jj}}}}$$

where c_jj_ is the jth diagonal element of the (X’X)^−1^ matrix, MSE_(i)_ is the mean squared error for the ith observation, ${\hat{\beta }}_{i}$ is the jth coefficient from the regression calculated using all data, ${\hat{\beta }}_{i,j}\;$ is coefficient from the regression calculated without the ith observation. A sample size-adjusted cut-off of $\pm \frac{3}{\sqrt{n}}$ (n is the number of runs) was assumed.

**Figure 11:**
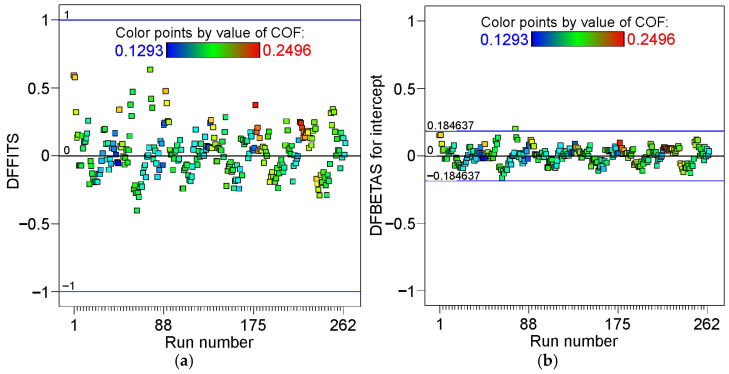
(**a**) Difference of fits vs. run number and (**b**) standardized difference of the betas vs. run number.

The graph showing the change in the DFFITS (Figure 11a) does not indicate the presence of outliers. A relatively large margin of error separating the points from the borderlines is also visible. In the case of the DFBETAS (Figure 11b), only one point can be considered a slight outlier.

Figure 10 shows response surface plots presenting the interaction between the Ra of the countersamples and the strip sample orientation affecting the COF. In general, the response surfaces for all the steel sheets analyzed are similar. As the average roughness Ra of the countersamples increases, the CoF decreases. Increasing the roughness of the harder tool relative to the sheet material reduces the real frictional contact area. Saha and Wilson [41] found that the CoF in strip friction tests decreased with a decrease in the contact area. Similar conclusions were reported by Hwang and Chen [42], who noted that a reduction in the real contact area leads to a decrease in the CoF.

Mousavi et al. [43] observed that the maximum friction force depends on the real contact area of the body.

**Figure 12:**
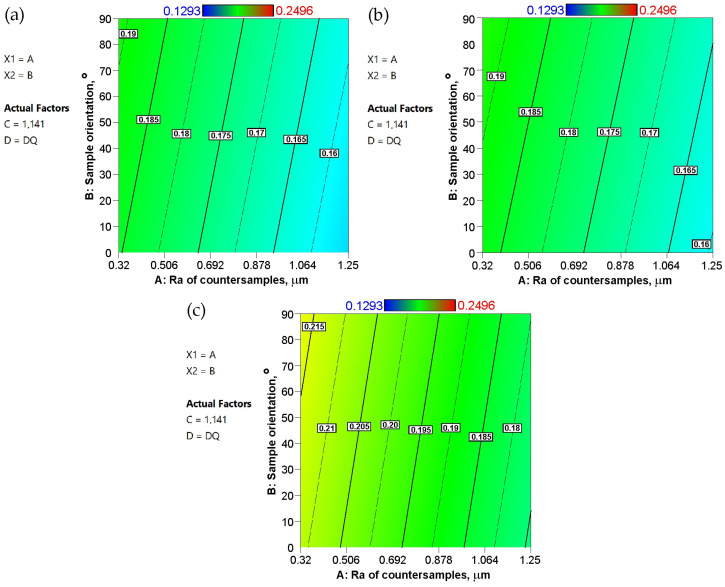
Response surface plots presenting the interaction between the Ra of countersamples and strip sample orientation affecting the COF for the (**a**) DQ steel sheet, (**b**) DDQ steel sheet, and (**c**) EDDQ steel sheet.

**Figure 13:**
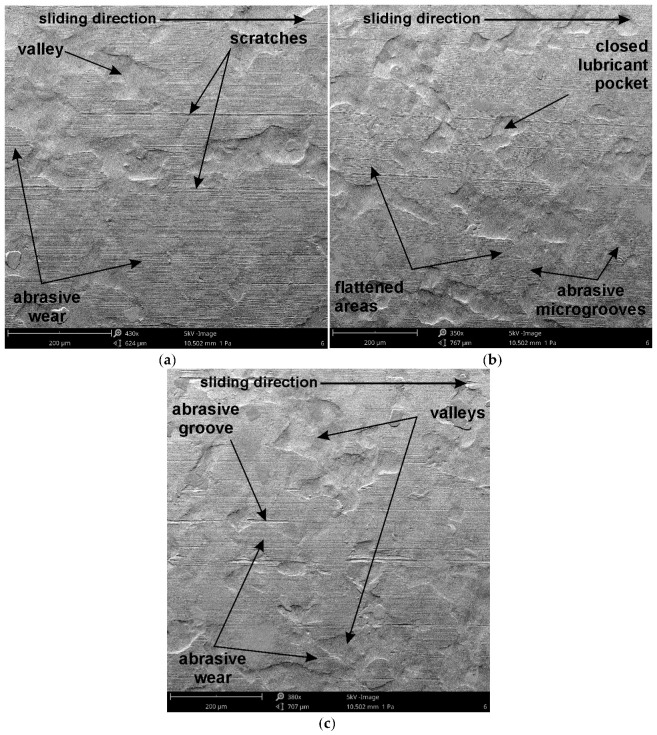
SEM images of worn surfaces of (**a**) DQ, (**b**) DDQ and (**c**) EDDQ samples (sample orientation, 0°; load force, 1.5 kN; roughness of countersamples, Ra = 0.63 μm).

As mentioned earlier, an increase in tool surface roughness reduces the real contact area. On the other hand, greater tool roughness also provides larger spaces between contacting surfaces that can accommodate lubricant, supporting the continuity of the lubricant film. In Figure 12, however, this difference for samples cut along the rolling direction is small—about 0.01 for the DQ (Figure 12a) and EDDQ (Figure 12c) steel sheets. This increase is independent of the average roughness of the countersamples. Across the entire range of input parameters, the EDDQ sheet exhibited a higher coefficient of friction (Figure 12c) compared with the other sheets (Figure 12a,b). Although the EDDQ sheet had the highest average roughness (Sa = 0.362), it also had the lowest yield strength. This means it is more prone to flattening asperity summits (Figure 13) under load, thereby increasing the contact area. On the surface of the tested sheets after the friction process, in addition to flattened asperities, grooves caused by the ploughing mechanism, scratches, and closed lubricant pockets can be observed (Figure 13). These pockets are beneficial under lubricated conditions because they retain lubricant, which, under load, acts as a pressure cushion transferring part of the load force.

**Figure 14:**
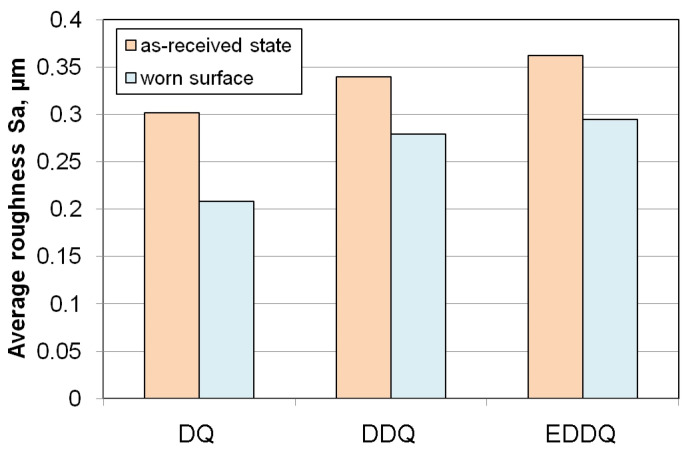
Friction-induced change in average roughness of sheet metals (sample orientation, 0°; load force, 1.5 kN; average roughness of countersamples, 0.63 μm).

The analysis of the surface roughness of the sheets presented in Figure 13 and the roughness of the sheets in the as-received state indicates friction-induced reductions in average roughness of 21.3%, 17.8%, and 31.2% (Figure 14) for the DQ, DDQ, and EDDQ sheets, respectively. The decrease in average roughness is an evident effect of the flattening of surface asperities shown in Figure 13. A quantitative evaluation of these roughness changes shows that the most significant reduction occurred in the sheet with the lowest yield strength and ultimate tensile strength. The EDDQ steel also exhibited the lowest tendency for strain hardening, as indicated by the value of the work-hardening exponent n. The remaining two grades (DQ and DDQ) demonstrated yield strengths at least 22% higher than that of the EDDQ sheet; therefore, they are less susceptible to friction-induced changes in average roughness under the same friction conditions.

It should be clearly emphasized that the strip drawing test is not a conventional wear test like the pin-on-disc test (Table 9). The strip drawing test reproduces friction conditions under the blank holder in SMF, where the friction path is non-repetitive and generally unidirectional, and the sliding distance does not exceed several tens of centimeters, even for large drawpieces. Under these conditions, there is no accumulation of wear products associated with a repeated friction path. The main friction mechanism in the strip drawing test is abrasive wear related to the flattening of sheet surface asperities (Figure 13). Abrasive friction occurs as a result of the mechanical flattening of the asperity peaks of one surface (the sheet) by the harder asperities of the counterface (tool). Only a few abrasive microgrooves were observed. Adhesive friction is associated with the formation of local microjunctions between asperities of two contacting surfaces. During relative motion, these junctions are sheared, leading to material transfer from one surface to the other. Under the investigated friction conditions, no signs of adhesion wear were observed. Escherová et al. [44] reported that the transition from adhesive to predominantly abrasive wear after quenching and tempering demonstrates the connection between increased hardness, microstructure (martensitic matrix + carbides), and CoF stabilization. The change in surface roughness observed in the friction testing of low-carbon steel sheets in the strip drawing test is a natural phenomenon, because in SMF, the strength of the tool material is intentionally higher than that of the sheet material.

**Figure 15:**
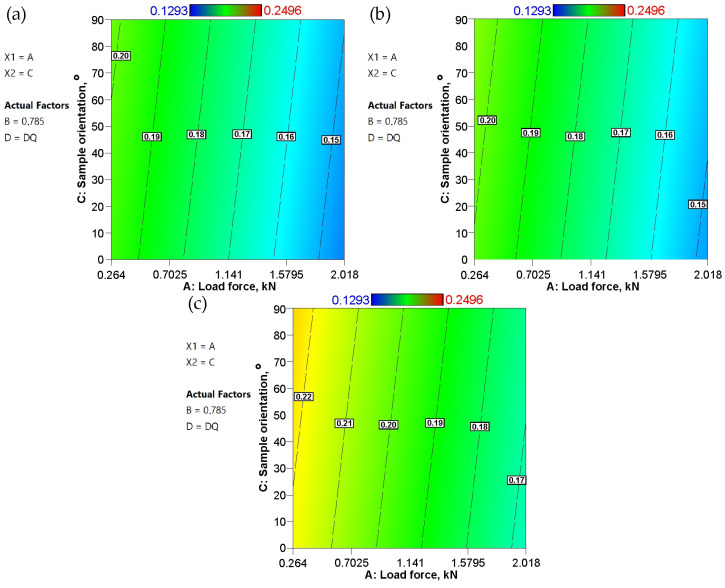
Response surface plots presenting the interaction between strip sample orientation and load force affecting the COF for the (**a**) DQ steel sheet, (**b**) DDQ steel sheet, and (**c**) EDDQ steel sheet.

The influence of sheet orientation on the coefficient of friction under conditions of variable load force is negligible (Figure 15). In contrast, the load force is the decisive parameter affecting the CoF. An increase in load force leads to a decrease in the CoF. This is typical behavior during strip drawing tests, where the tested sheet has significantly lower hardness than the tool material. Under such conditions, plastic deformation of asperity summits and mechanisms such as flattening and ploughing determine resistance to friction. The observed decrease in the CoF with an increase in load was explained by Vollertsen and Hu [20] and Sigvant et al. [45] to be a lack of proportionality between friction force and normal force when metallic contact between the surfaces is dominant. The nonlinearity between friction, normal load, and real contact area was also reported by Kalin and Jan [46]. Moreover, the CoF is higher for contact with a smooth surface at low normal forces but decreases as the normal force increases [47].

**Figure 16:**
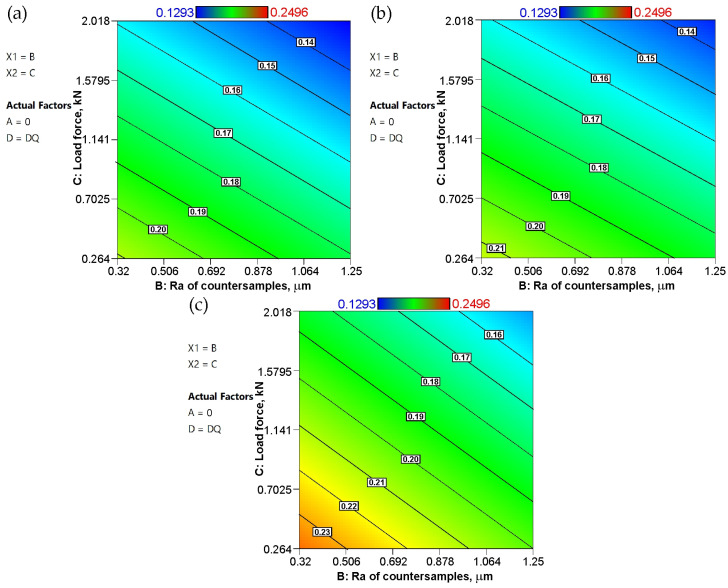
Response surface plots presenting the interaction between the Ra of the countersamples and load force affecting the COF for the (**a**) DQ steel sheet, (**b**) DDQ steel sheet, and (**c**) EDDQ steel sheet.

The response surfaces of the combined influence of load force and the average roughness of countersamples on the CoF are more complex. The lowest coefficient of friction was observed for the highest values of these parameters (Figure 16). On the other hand, friction under conditions of low load force and low countersample roughness resulted in the highest CoF. This trend applies for all the steel sheet grades investigated. Considering the slope of the surfaces relative to the coordinate planes, the load force has a greater effect on the CoF of the sheets than the average roughness of the countersamples.

Although only one type of oil was considered in this study, it should be noted that the lubrication conditions during SMF depend on the viscosity of the oil. Oil with higher viscosity is more effective in reducing the CoF compared to oil with lower viscosity, as observed by Lee et al. [29]. Shisode et al. [48] concluded that with an increase in lubricant viscosity, lubricant pressure increases. Oils with higher viscosity form a thicker and more durable lubricant film, which more effectively separates the metal and tool surfaces. Interactions between the surface asperities of the sheet and the tool lead to a range of contact phenomena that directly affect friction, wear, and the surface quality of the finished component. These interactions can be considered at both the micro- and macroscopic levels, taking into account both the surface topography and mechanical properties [4,22]. As the load increases, the asperity summits undergo elastic deformation (at low loads), plastic deformation, or shearing. Under the influence of the tool load, the sheet asperities flatten, which increases their load-bearing area and reduces the initial roughness but, at the same time, also increases the contribution of adhesion to the total friction resistance [49]. Plastic deformation, especially in low-carbon steel sheets, is accompanied by the work-hardening phenomenon, which increases the material strength of the sheet due to a strain-induced rise in dislocation density. The surface asperities of the sheet determine the way loads are transferred and the contribution of adhesive, abrasive, and mixed friction [50].

The alignment of crystalline grains in sheet metals formed during the rolling process plays a key role in how a metal sheet behaves under stress [51]. Rolled low-carbon steels exhibit pronounced planar anisotropy resulting from a rolling-induced crystallographic texture. Typical texture components in these materials include a γ-fiber ({111}) and α-fiber (<110>), which influence plastic deformation behavior depending on the orientation relative to the rolling direction [52]. Consequently, mechanical properties and material flow may differ between specimens oriented parallel and perpendicular to the rolling direction [53,54]. This effect was also observed in the present study, as indicated by the differences in the tensile test results for the 0° and 90° orientations (Table 1). Such an anisotropic plastic response can affect the real contact conditions and consequently the friction coefficient measured in strip drawing tests.

The results of the variance analysis presented in this paper demonstrated the influence of sample orientation on changes in the CoF of low-carbon steel sheets in the as-received condition. The effect of rolling-induced surface texture and anisotropy of mechanical properties on the friction phenomenon is most pronounced under large deformations. Therefore, future studies should consider evaluating the frictional behavior of pre-stretched strip samples. Pre-deformation of the sheets will alter both the surface topography and increase the strength of the sheet while reducing its plasticity.

## 4. Limitations of This Work

When developing a friction model using analysis of variance, the influence of contact force on phenomena occurring in the contact zone was considered. A more practical indicator explaining contact phenomena would be a non-dimensional parameter, such as the ratio of contact pressure p_n_ to yield strength σ_y_. However, in the present strip drawing configuration, the contact between the cylindrical tool and the flat strip results in a non-uniform pressure distribution along the contact arc. A single representative contact pressure value is not directly measurable and can only be approximated under simplifying assumptions. Furthermore, under the conditions investigated, the surface asperities undergo local plastic deformation and strengthening accompanied by work hardening, leading to a continuously evolving local yield strength at the contact interface. The influence of local deformations on the change in the yield strength of asperities in rough contact due to the work-hardening phenomenon is rather difficult to assess analytically and quantitatively. Therefore, defining a unique and physically representative value of p_n_/σ_y_ for the contact zone is not straightforward in elstic–plastic contact. It should also be noted that the friction coefficient itself is a non-dimensional quantity (the ratio of tangential to normal force), which inherently reflects the influence of the normal load applied.

## 5. Conclusions

This paper presents the results of friction tests conducted on low-carbon steel sheets using the strip drawing test, which is used to evaluate sheet friction in metal-forming operations. The analysis of the relationships between the strip drawing test parameters and CoF allowed us to draw the following conclusions:

1. Load force was the parameter that most strongly influenced changes in the coefficient of friction. However, the effect of sheet orientation on the CoF under a varying load force was small.
2. Strip samples cut transversely to the rolling direction of the sheet exhibited a higher coefficient of friction, in accordance with the response surfaces.
3. The effect of the average roughness of countersamples on the CoF is more complex. At the highest roughness combined with the highest load force, the CoF reached its lowest values. At a constant load force, the CoF increased as the average roughness of the countersamples decreased.

In SMF, the contact force significantly affects friction by altering the real contact conditions between the tool and the sheet. Under lubricated conditions, higher contact force may enhance lubricant retention in surface valleys. Therefore, friction plays a critical role in controlling material flow and overall process stability in deep-drawing operations. Analysis of sheet orientation relative to the rolling direction is significant in practice because sheet metals are typically produced via rolling processes, which induce anisotropic material properties and directional surface topography. Although the influence of orientation on the CoF was found to be relatively small under varying load forces, the higher CoF observed for transverse samples may contribute to non-uniform material flow and should be considered in precision forming applications. Under industrial conditions, tool surfaces progressively change due to wear or surface degradation. The finding that higher roughness combined with higher load resulted in a lower CoF suggests the presence of lubrication retention or altered real contact area mechanisms, which may be beneficial in certain operating regimes.

If low frictional resistance is required under lubricated conditions in the blank holder zone, tools with high average surface roughness and high contact pressure should be applied. Intermediate friction conditions can be achieved by integrating a high average roughness of the countersamples with low load force or low average roughness of the countersamples with a medium load force. The influence of these two parameters on the CoF results from the combined effect of load force, the elastic–plastic deformation behavior of the sheet surface asperities, and lubricant retention and behavior within the oil pockets. These conclusions apply to all sheet grades tested and the entire range of variability in the friction process parameters used in the experimental study.

Analysis of the ANOVA model allowed the following conclusions to be drawn:

1. The adequate precision of the ANOVA model, equal to approximately 104.7431, and the coefficient of determination R^2^ = 0.9367 indicated that the regression model was sufficiently fitted to provide reliable predictions. The high predictive quality of the model was also confirmed by the small difference (0.0019) between the adjusted and predicted R^2^.
2. The model-derived F-value of 541.60 indicates the significance of the determined mathematical model. All friction process parameters (sample orientation, surface roughness of countersamples, normal load, and type of sheet metal) were found to be significant at a 95% confidence level.
3. Analysis of Cook’s distance showed that there were no points significantly deviating from the rest of the data, meaning that all measurement points had a significant influence on the model response.

## Figures and Tables

**Table 1:** The basic mechanical parameters of the steel sheets.

Parameter	EDDQ		DDQ		DQ	
	0	90	0	90	0	90
Elongation A, %	44	42	42	41	36	34
YS, MPa	151	153	196	198	193	193
UTS, MPa	282	287	336	311	351	353
K, MPa	494	487	557	526	554	563
n	0.22	0.21	0.19	0.18	0.17	0.17

**Table 2:** The surface roughness parameters of the tested sheets.

Parameter	EDDQ	DDQ	DQ
Sq, μm	0.41	0.423	0.376
Sa, μm	0.362	0.340	0.302
Sku	3.67	3.34	3.48
Ssk	0.338	0.298	0.267

**Table 3:** The surface roughness parameters of the countersamples.

Ra, μm	Sq, μm	Sa, μm	Sku	Ssk
0.32	0.56	0.43	8.43	−0.93
0.63	0.72	0.56	4.11	−0.27
1.25	1.57	1.34	3.01	−0.29

**Table 4:** Statistics of the input variables.

Factor	Name	Unit	Type	Subtype	Min.	Max.	Coded Low	Coded High	Mean	Std. Dev.
A	Sample orientation	°	Numerical	Continuous	0.0000	90.00	−1 ↔ 0.00	+1 ↔ 90.00	45.34	45.08
B	Ra of countersamples	μm	Numerical	Continuous	0.3200	1.25	−1 ↔ 0.32	+1 ↔ 1.25	0.7286	0.3877
C	Normal load	kN	Numerical	Continuous	0.2640	2.02	−1 ↔ 0.26	+1 ↔ 2.02	1.12	0.5325
D	Type of sheet metal	-	Categorical	Nominal	DQ	EDDQ			Levels:	3

**Table 5:** Statistics pertaining to the response variable.

Response	Name	Unit	Number of Observations	Min.	Max.	Mean	Standard. Deviation
R1	CoF	-	264	0.1293	0.2496	0.1841	0.0239

**Table 6:** Correlation between input variables and CoF.

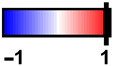	Run	A	B	C	D	CoF
Run	1	0.293	0.146	0.044	-	0.291
A: Sample orientation	0.293	1	0.004	0.006	-	0.124
B: Mean roughness of countersamples, Ra	0.146	0.004	1	−0.009	-	−0.546
C: Normal load, F_N_	0.044	0.006	−0.009	1	-	−0.655
D: Grade of steel sheet metal	-	-	-	-	-	-
Coefficient of friction	0.291	0.124	−0.546	−0.655	-	1

**Table 7:** Results of ANOVA for the response surface of the polynomial 2FI model.

Source	Sum of Squares	Degree of Freedom	Mean Square	F-Value	*p*-Value	
Model	0.1410	7	0.0201	541.60	<0.0001	significant
A—Sample orientation, °	0.0024	1	0.0024	64.10	<0.0001	
B—Ra of countersamples, μm	0.0457	1	0.0457	1229.07	<0.0001	
C—Normal load, kN	0.0650	1	0.0650	1746.54	<0.0001	
D—Sheet metal	0.0274	2	0.0137	367.67	<0.0001	
BD	0.0007	2	0.0004	9.67	<0.0001	
Residual	0.0095	256	0.0000			
Correlation total	0.1506	263				

**Table 8:** Fit statistics of the 2FI model.

Standard deviation	0.0061	R^2^	0.9367
Mean	0.1841	Adjusted R^2^	0.9350
Coefficient of variation, %	3.33	Predicted R^2^	0.9331
		Adequate precision	104.7431

**Table 9:** Differences between the strip drawing test and pin-on-disc tests conducted at ambient temperature.

Characteristics	Strip Drawing Test	Pin-on-Disc Test
type of test	friction test	wear test
main task	quantitative determination of CoF	quantitative determination of wear
the length of the friction track	usually does not exceed several centimeters	unlimited
repeatability of contact	no	yes
recirpocating sliding	no	possible, depending on the approach
friction-induced heating	very low	very high
sliding speed	0.1–50 mm/s	0.01–10 m/s

## Data Availability

The original contributions presented in this study are included in the article. Further inquiries can be directed to the corresponding author.
